# Editor’s introduction to this issue (G&I 19:2, 2021)

**DOI:** 10.5808/gi.19.2.e1

**Published:** 2021-06-30

**Authors:** Taesung Park

**Affiliations:** Department of Statistics, Seoul National University, Seoul 08826, Korea

In this issue, there are six original articles and one mini review. The first article by by Sohag et al. (Jagannath University, Bangladesh) provides a short review on omics approaches to cardiovascular diseases (CVDs). The author summarizes the genomics, proteomics, transcriptomics, and metabolomics in CVDs with a well-organized prospect.

The first original article is about a protein interactions map of multiple organ systems associated with coronavirus disease 2019 (COVID-19) disease by Dr. Bharne (University of Hyderabad, India). This study appears to be motivated by reports that reduced antibody levels and disease recurrence in recovered COVID-19 patients require understanding of the epidemic at a key level. Multiple organ failure cases in patients with COVID-19 have highlighted consideration for other organ systems. This study used RNA sequencing data to determine disease-associated differentially regulated genes and related protein interactions in multiple organ systems, which implies the importance of early diagnosis and treatment of the disease. RNA sequencing data were obtained from autopsy specimens of lung, heart, jejunum, liver, kidney, intestine, bone marrow, adipose, placenta, and skin from 24 patients who died of COVID-19 infection. The total number of samples in the sequencing data was 88, including five negative control samples. Using significantly expressed genes in different organ systems, protein interactions of multiple organ systems were then mapped, revealing CAV1 and CTNNB1 as top nodes. A core interactions sub-network was analyzed to identify several functionally important modules such as AR, CTNNB1, CAV1 and PIK3R1 proteins. In addition, this study highlighted some of the druggable targets to analyze in drug re-purposing strategies against the COVID-19 pandemic. I think the protein interaction maps and modular interactions of differentially regulated genes in multi-organ systems would provide the clues to researchers to rapidly investigate novel therapeutics for the COVID-19 pandemic.

The second article by Sohpal (Beant College of Engineering & Technology, India) performed a comparative study of coronaviruses including severe acute respiratory syndrome coronavirus 2, severe acute respiratory syndrome coronavirus, and Middle East respiratory syndrome coronavirus focusing on non-synonymous and synonymous substitutions Through simulation studies, nucleotide sequence of closely related strains of respiratory syndrome viruses, codon-by-codon with maximum likelihood analysis, z selection and the divergence time were investigated.

The third article by Mahfuz et al. (University of Development Alternative, Bangladesh) presented a network-biology approach for identification of key genes and pathways involved in malignant peritoneal mesothelioma (MPM). To understand the molecular mechanisms responsible for the initiation and progression of MPM, this study aims to identify the key genes and pathways responsible for MPM. Several bioinformatics analyses were performed such as identification of differentially expressed genes, pathway analysis, and protein-protein interaction network analysis, providing an insight into the potential genes and pathways involved in MPM.

The next article by Kotipalli et al. (Centre for Development of Advanced Computing, India) presents the results of epigenetic analysis for breast cancer. Using chromatin immunoprecipitation sequencing, epigenetic regulation of gene expression by in-silico analysis of histone modifications was carried out. Histone modification data of H3K4me3 from one normal-like and four breast cancer cell-lines were used to predict miRNA expression at the promoter level. Predicted miRNA promoters were used as a probe to identify gene targets. This study is expected to provide the insight into predicted role of H3K4me3 mediated gene regulation via the miRNA-mRNA relationship.

The next article by Oh (Inje University College of Medicine, Korea) is about G protein‒coupled receptors, including olfactory receptors. Through simulation study, the interaction between human olfactory receptor 1A1 and the corresponding odorant molecule was investigated. Further, it was also studied how the chemically simple odorant molecule activates the olfactory receptor.

Finally, the last original article by Choi’s group (Kangwon National University, South Korea) presents analysis of genome variants in dwarf soy­bean lines obtained in F6 derived from cross of normal parents (cultivated and wild soybean). Through whole genome sequencing data analysis, the authors reported DNA variations between the normal and dwarf members of four lines harvested from a single seed parent in an F6 recombinant inbred line population. The list of single nucleotide polymorphisms provides important informa­tion for the genetics of soybean plant height and crop breeding and expected to be useful genetic resources for plant breeders.

